# Impacts of off-flavor on microbial community structure, nutritional traits and metabolite profiles in whole-plant corn silage

**DOI:** 10.3389/fmicb.2026.1827817

**Published:** 2026-04-30

**Authors:** Zihui Wang, Xiaomiao Guo, Yining Shi, Ning Ma, Yufei Ma, Yushuang Pei, Yingmin Li, Hongjian Xu, Fengtao Ma, Yan Li, Jianguo Li, Yanxia Gao

**Affiliations:** 1 College of Animal Science and Technology, Hebei Agricultural University, Baoding, China; 2 College of Veterinary Medicine, Hebei Agricultural University, Baoding, China; 3 Key Laboratory of Healthy Dairy Cattle Breeding of the Ministry of Agriculture and Rural Affairs (Co-Constructed by Ministry and Province), Baoding, China; 4 Hebei Innovation Center of Cattle and Sheep Embryo Technology, Baoding, China; 5 Hebei Research Institute of Dairy Industry Technology, Shijiazhuang, China

**Keywords:** corn silage, flavor, metabolites, phenylacetic acid, silage quality

## Abstract

Off-flavor in corn silage compromises feed quality and animal performance, yet the microbial-metabolic changes driving its formation remain poorly understood. In this study, microbiome profiling, metabolomics, and sensory evaluation were integrated to elucidate the changing factors driving off-flavor formation. Silage samples from 10 dairy farms were classified by sensory score into control (CON, 15–20) and abnormal silage groups (ASG, <15). The bacterial community structure was analyzed, metabolites were identified and quantified using gas chromatography-mass spectrometry (GC-MS), and the associations between microorganisms and metabolites were explored via Spearman correlation analysis. Compared with the CON, the ASG exhibited significantly lower concentrations of dry matter (*p* = 0.01), starch (*p* = 0.04), lactic acid (*p* = 0.03), and acetic acid (*p* = 0.04), while those of neutral detergent fiber (*p* = 0.02), acid detergent fiber (*p* = 0.03), propionic acid (*p* = 0.01), and butyric acid (*p* = 0.02) were significantly higher. The pH value of the ASG was increased to 4.99 compared to that of the CON (3.74), with a significant difference observed between them. Meanwhile, the abundances of phylum Firmicutes (p-Firmicutes) and genus Lactobacillus (g-Lactobacillus) in the ASG were both significantly lower than CON. After further screening from the 32 differentially abundant metabolites (excluding 16 redundant metabolites and 2 that remained unannotated due to database limitations), a total of 14 core differential metabolites associated with off-flavor in silage were identified. Among these, compounds contributing acetic and waxy aromas were enriched in the ASG. Spearman correlation analysis showed *Corynebacterium* and *JC017* were positively correlated with fatty and waxy compounds (methyl linoleate, oleic acid), while Massilia and *Paenibacillus_D* were negatively correlated with phenylacetic acid. These findings demonstrate that off-flavor in corn silage arises from Lactobacillus decline, aerobic spoilage bacteria overgrowth, and subsequent accumulation of phenylacetic acid coupled with gallic acid depletion. This microbiome-metabolomics interaction provides a mechanistic framework for developing targeted strategies to control silage off-flavors.

## 1 Introduction

The global dairy industry is undergoing rapid large-scale restructuring, with silage emerging as a core component of cattle rations due to its high biomass yield and efficient nutrient utilization. As a key preservation technique for silage, anaerobic fermentation effectively retains nutrients and improves feed palatability. However, inadequate airtight sealing during ensiling or secondary fermentation during storage can cause flavor deterioration (da Silva et al., 2020).

Silage off-flavors are primarily induced by microbial community dysbiosis throughout the fermentation process, in conjunction with improper operational practices during silage fermentation, storage, and utilization (Raza et al., 2016; Robinson and Swanepoel, 2016). At the microbial community level, lactic acid bacteria affiliated with the phylum Firmicutes have been identified as the dominant functional microbes driving silage fermentation (da Silva et al., 2020); a decrease in their abundance leads to insufficient lactic acid production, which in turn increases silage pH and fosters the proliferation of harmful microorganisms. Specifically, excessive growth of Clostridia can induce the conversion of carbohydrates to butyric acid in alfalfa silage, a key driver of flavor degradation, as reported by Li et al. (2020). Additionally, Robinson and Swanepoel (2016) found that the air-exposed outer surfaces of silage bales exhibit a markedly higher propensity for microbial spoilage, leading to the production of pungent, moldy odors in haylage. Furthermore, the high contents of starch, protein, fat, and tannins in sweet sorghum provide optimal metabolic substrates for brewing yeast, whose two key anaerobic fermentation pathways mediate aroma modifications in silage (Li et al., 2021).

These microbial activities directly shape the metabolic profile of silage, which is closely linked to sensory quality and animal health. The volatile compounds in whole-plant corn silage are predominantly composed of alcohols, short-chain organic acids, esters, aldehydes, ketones, and phenolic-phenylpropane derivatives, whose compositional characteristics and relative abundances directly determine silage sensory quality (Hafner et al., 2013). Although Chen et al. (2025) characterized the volatile organic compounds (VOCs) of whole-plant corn silage using a variety of detection techniques, their study lacked an in-depth investigation into the targeted correlations between specific microbial taxa and off-flavor metabolites. Beyond off-flavors, mycotoxin contamination-a serious metabolic consequence of microbial dysbiosis-exerts adverse effects on dairy cow health. Rocchetti et al. (2021) demonstrated that mycotoxin-contaminated corn silage can induce systematic alterations in the metabolomic profiles of dairy cow milk, with distinct variation patterns associated with different mycotoxin classes, including aflatoxins and fusarium toxins. In sweet sorghum silage, a pronounced wine-like aromatic enhancement has been observed, characterized by the biosynthesis of ethyl acetate and an increase in ethanol content (Li et al., 2021). Based on these findings, we hypothesize that specific microbial communities and their corresponding off-flavor metabolites collectively drive the sensory quality alterations of silage during fermentation.

Existing studies have demonstrated that characteristic flavor compounds in silage are mostly classified into specific categories, such as phenols and aldehydes. However, the flavor changes induced by individual inherent or metabolically generated compounds in silage have not yet been clearly investigated. Therefore, the present study was designed to analyze the correlation between microbial community structure and metabolites in silage, and to screen for the key compounds that may be responsible for flavor alterations.

## 2 Materials and methods

### 2.1 Experimental materials

A total of 20 silage samples (2 independent samples per farm) were collected from silos on 10 large-scale dairy farms in Hebei Province, covering Shijiazhuang (6 farms), Baoding (2 farms), and Xingtai (2 farms). Sampling was performed according to the method described by Chen et al. (2025). The cross-section of the silo was equally divided into nine sampling points, and a circle with a radius of 50 cm was drawn around each point as the center. Silage samples were collected at a depth of 20 cm at each sampling circle, thoroughly mixed, and reduced to 500 g using the quartering method (Chen et al., 2025). The prepared samples were placed into polyethylene silage bags and vacuum-sealed using a vacuum packaging machine, followed by transportation under cold chain conditions. All samples were stored in dry ice-cooled foam boxes for subsequent analysis.

### 2.2 Experimental design

A single-factor experimental design was adopted in this study. Silage samples were collected from 10 dairy farms, with two independent samples harvested from each farm-one from the central area and the other from the peripheral area of a single silo. All collected samples were subjected to sensory evaluation by six professionally trained panelists, and the two silage samples from each farm were subsequently classified into the control group (CON, sensory score: 15–20) and the abnormal silage group (ASG, sensory score: <15), resulting in a total of 20 original silage samples for the study. Silage sensory evaluation followed the protocol of the German Agricultural Society (Zhu et al., 1999; see Supplementary Table 1). Quantitative scoring was conducted for three key parameters-color, odor, and texture.

### 2.3 Silage nutritional components and quality assessment

Silage samples were dried at 55 °C for 48 h until a constant weight was attained, which were then prepared for subsequent analysis. Dry matter (DM), crude protein (CP, Kjeldahl method), neutral detergent fiber (NDF) and acid detergent fiber (ADF) were determined according to the AOAC standard methods (Lee, 1995). The contents of NDF and ADF were determined using a fiber analyzer (A200I, ANKOM, Macedon, NY, United States). For fermentation profiling, 20 g of fresh silage was mixed with 180 mL distilled water in polyethylene bags. The mixture was incubated at 4 °C for 24 h. A portable pH meter (Shanghai Yidian Science and Technology Co., Ltd., PHS-25, Shanghai, China) was used to measure the pH value of silage samples. The supernatant was filtered and analyzed for NH_3_-N (determined by phenol-hypochlorite colorimetry using UNICO UV-2102 PCS spectrophotometer), and volatile fatty acids (VFAs, including acetic, propionic, and butyric acids) using an ultra-high performance gas chromatography system (Laboratory of Beijing University of Chinese Medicine). The lactic acid content was determined using a commercially available assay kit (Nanjing Jiancheng Bioengineering Institute, Nanjing, China).

### 2.4 DNA extraction and 16S rRNA gene sequencing and analysis

Total genomic DNA was extracted from the samples with the MagBeads FastDNA Kit for Soil (Cat. No. 116564384, MP Biomedicals, Santa Clara, CA, United States) in strict accordance with the manufacturer’s protocols, and the extracted DNA samples were stored at −20 °C for subsequent analysis. The quantity and quality of extracted DNA were measured using a NanoDrop NC2000 spectrophotometer (Thermo Fisher Scientific, Waltham, MA, United States) and agarose gel electrophoresis, respectively. PCR amplification of the bacterial 16S rRNA genes V3-V4 region was performed using the forward primer 338F (5′-ACTCCTACGGGAGGCAGCA-3′) and the reverse primer 806R (5′-GGACTACHVGGGTWTCTAAT-3′).

Alpha diversity index analysis: Five diversity indices were calculated for each sample using QIIME2 software, including the Chao1 (Chao 1984), observed, Shannon (Shannone 1948), Simpson (1949) and observed-species. Beta diversity analysis was performed based on the UniFrac distance metric to investigate variations in microbial community structure among samples, and the results were visualized via principal coordinates analysis (PCoA). Based on correlation coefficients and significance levels, a modular network was built, with nodes representing ASVs and edges indicating inter-ASV correlations. Visualization was conducted using R and the ggraph package.

### 2.5 Analysis of metabolites present in silage samples

#### 2.5.1 Sample preparation and treatment

Silage samples (50 ± 1 mg) were weighed into 2 mL EP tubes, with 500 μL pre-chilled extraction solvent (methanol: water, 3:1, v/v, containing ribitol) added. The mixture was vortexed for 30 s; then stainless steel beads were introduced, and the samples were ground at 40 Hz for 4 min, followed by three cycles of ice-water bath sonication (5 min each). Samples were centrifuged at 13800×g and 4 °C for 15 min, and 200 μL supernatant was transferred to 1.5 mL EP tubes. A 50 μL aliquot of each supernatant was pooled to prepare quality control (QC) samples. Dried extracts were reconstituted with 50 μL methoxyamine reagent (20 mg/mL in pyridine) and incubated at 80 °C for 30 min, then derivatized with 70 μL BSTFA (1% TMCS, v/v) at 70 °C for 1.5 h. After cooling, 5 μL FAMEs (in chloroform) was added, and all samples were analyzed in a random sequence by GC.

#### 2.5.2 GC-MS analysis

Gas Chromatography-Mass Spectrometry (GC-MS) analysis was performed using a SHIMADZU GC-2020 gas chromatograph (30 m × 250 μm × 0.25 μm, J&W Scientific, Folsom, CA, United States) coupled with a mass spectrometer (Model QP2020 NX, Shimadzu, Tokyo, Japan). The system was equipped with a DB-5MS capillary column. A 1 μL aliquot of the sample was injected in splitless mode. Helium was employed as the carrier gas; the front inlet purge flow was set at 3 mL·min^−1^, and the column flow rate was maintained at 1 mL·min^−1^. The initial column temperature was held at 50 °C for 1 min, then was ramped up to 310 °C at a rate of 8 °C·min^−1^, and finally was held at 310 °C for 11.5 min. The injection port, transfer line, and ion source temperatures were set at 280 °C, 280 °C, and 200 °C, respectively. Ionization energy was set at −70 eV in electron impact (EI) mode. Mass spectrometry data were acquired in full-scan mode after a solvent delay of 7.2 min, with an *m*/*z* range of 50–500 and a data acquisition rate of 12.5 spectra per second.

### 2.6 Statistical analysis

Data processing for the present study was conducted using SPSS 27.0 (SPSS Inc., Chicago, IL, United States). For the 20 independent silage samples (2 samples per farm, divided into CON and ASG groups), 3 technical replicates were set for each sample to determine the nutritional and fermentation quality indicators, aiming to eliminate random errors caused by experimental operations and instrument detection. Subsequent statistical analyses were performed using a paired *t*-test to assess the statistical significance of differences between paired groups. Experimental data are expressed as means and standard errors of the mean (SEM). Statistical significance was determined based on *p*-values, with *p* < 0.05 considered statistically significant. This threshold was applied to analyze the nutritional components and volatile fatty acids (VFAs) of whole-plant corn silage.

Considering the high sample consumption and associated detection costs of high-throughput sequencing and metabolomic analysis, 10 biological replicates were set for each group, and each biological replicate was subjected to two technical replicates, resulting in a total of 40 assay units (i.e., 20 per group). Microbial community data were analyzed using the Personalbio Cloud Platform^1^, an online bioinformatics analysis tool, for the statistical and bioinformatic assessment of 16S rRNA gene sequencing datasets, including analyses of alpha diversity, beta diversity, and variations in microbial community composition.

Metabolite data were also analyzed using the Personalbio Cloud Platform. Differential metabolites in silage were screened against the thresholds of *p* < 0.05, fold change (FC) >2.00 or <0.5, and variable importance in projection (VIP) >1. Subsequent metabolic pathway enrichment analysis and microbe-metabolite correlation analysis were also conducted on this platform. It should be noted that the 20 independent silage samples served as the core biological replicates in this study (2 biological replicates per farm). All 40 samples used for microbial and metabolomic analyses were derived from these 20 core biological samples, which guaranteed full consistency and traceability across the entire experimental dataset.

## 3 Results

### 3.1 Nutritional component differences between silage groups

The nutritional characteristics of two-group silage are shown in Table 1. The contents of crude protein (*p* = 0.21), crude fat (*p* = 0.11) and crude ash (*p* = 0.07) remained stable, with no significant differences observed between the two groups. Compared with the CON group, the contents of dry matter (*p* = 0.01), starch (*p* = 0.04) and acetic acid (*p* = 0.04), were significantly decreased in the ASG group, with a marked reduction in lactic acid content (*p* = 0.03). The contents of neutral detergent fiber (*p* = 0.02), the ratio of ammonia nitrogen to total nitrogen (*p* = 0.02), acid detergent fiber (*p* = 0.03), propionic acid (*p* = 0.01), and butyric acid (*p* = 0.02) were significantly higher than CON. The pH values of the two silage groups were observed to fluctuate between 3.74 and 4.99, with significant differences being noted (*p* = 0.03).

**Table 1:** Nutritional differences in two-group silage (*n* = 10).

Items%	Groups		SEM	*p*-value
	CON	ASG		
DM	37.30	29.45	0.42	0.01
CP	8.27	8.11	0.13	0.21
EE	3.53	3.69	0.09	0.11
Ash	5.12	4.86	0.14	0.07
NDF	41.21	46.72	0.48	0.02
ADF	21.70	27.23	0.30	0.03
Starch	36.22	31.29	0.16	0.04
pH	3.74	4.99	0.02	0.03
AN/TN	8.98	11.52	0.24	0.02
LA	5.3	4.27	0.22	0.03
AA	1.30	1.01	0.05	0.04
PA	0.11	0.37	0.01	0.01
BA	0.02	2.10	0.16	0.02

Ten paired samples were obtained from the con and ASG groups, respectively, (*n* = 10), yielding a total of 20 samples, with three technical replicates performed for each sample. DM, dry matter; CP, crude protein; EE, crude fat; Ash, coarse ash content; NDF, neutral detergent fiber; ADF, acid detergent fiber; AN/TN, ammonia nitrogen/total nitrogen; LA, lactic acid; AA, acetic acid; PA, propionic acid; BA, butyric acid.

### 3.2 The impact of silage microbial community

#### 3.2.1 Analysis of microbial diversity in silage

**Figure 1:**
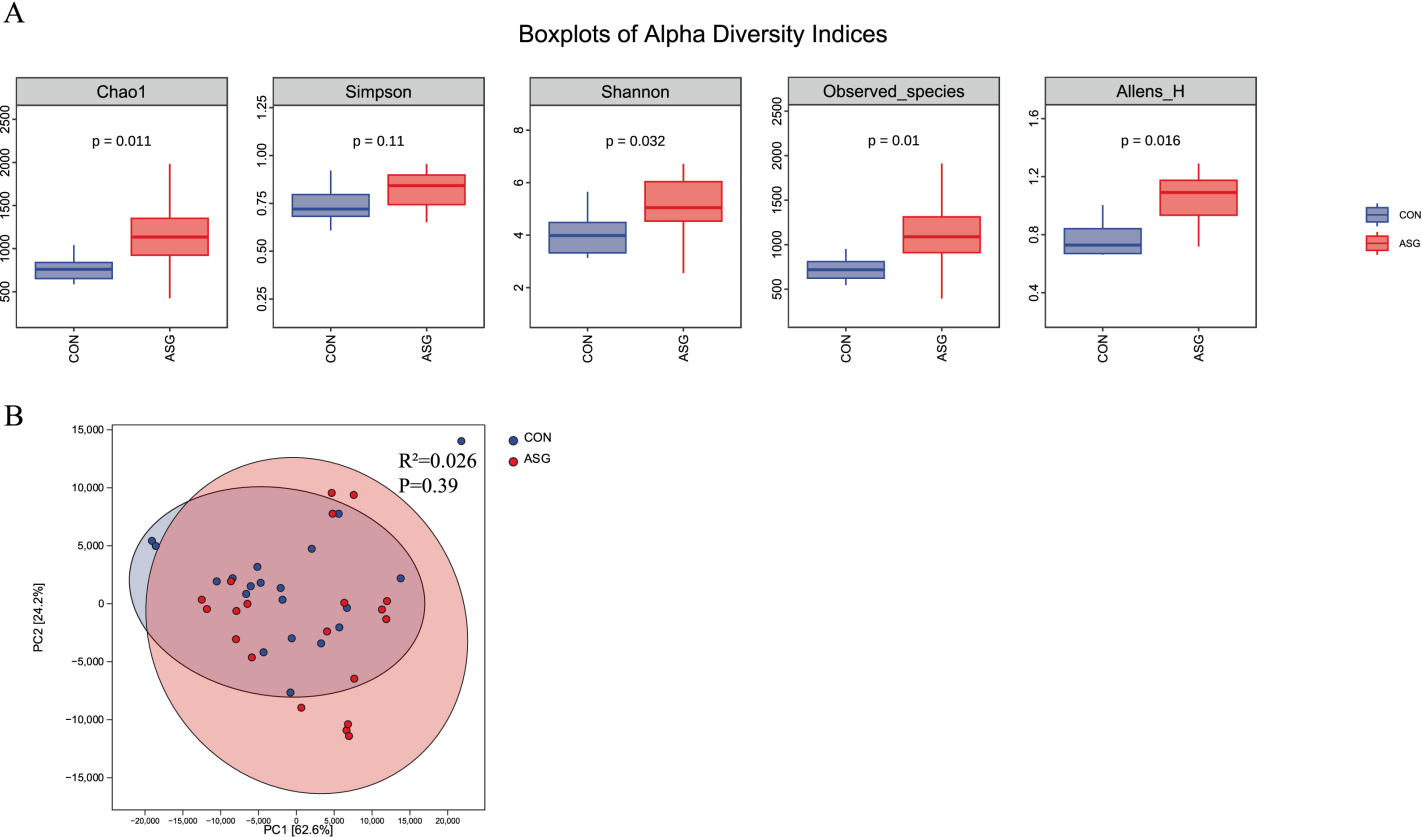
**(A)** Alpha diversity indices of silage bacteria, **(B)** beta-diversity analysis of silage bacteria.

As shown in the alpha diversity analysis (Figure 1A), the Chao1, Shannon, Observed_species, and Allens_H indices of the ASG were significantly higher than those of the CON (*p* < 0.05). The Simpson index also showed an increasing trend (*p* = 0.11), indicating an improvement in community evenness. The PCoA plot (Figure 1B) showed no clear separation between the CON and ASG samples in the ordination space.

#### 3.2.2 Differential analysis of microbial phylum and genus levels in silage

**Figure 2:**
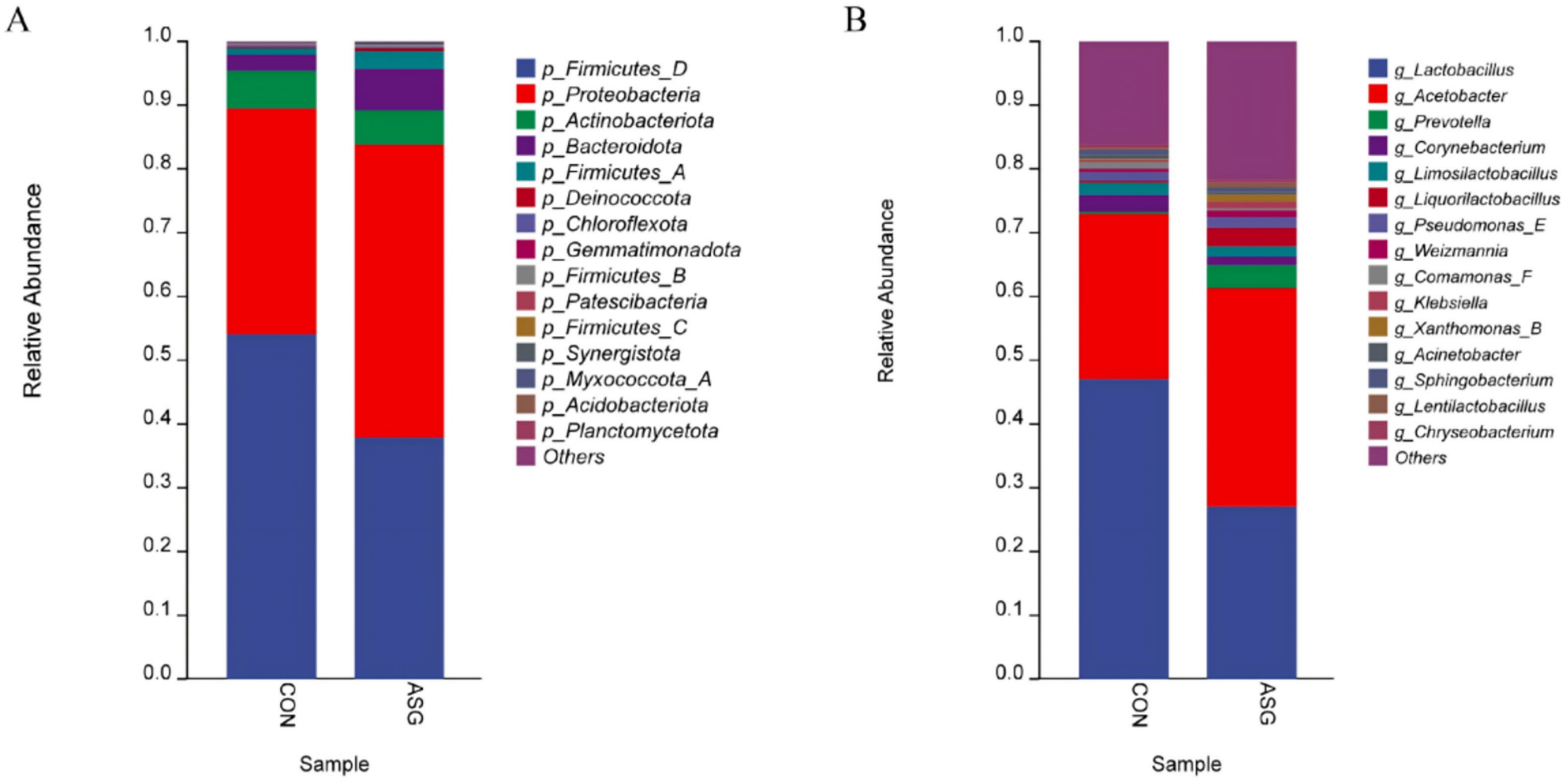
**(A)** Phylum-level composition and **(B)** genus-level composition.

A total of 15 bacterial phyla were identified. Among these, five dominant phyla were Firmicutes_D (54%), Proteobacteria (35%), Actinobacteriota (6%), Bacteroidota (2.4%), and Firmicutes_A (1%), as presented in Figure 2A. Meanwhile, compared with the CON, the relative abundance of Actinobacteriota and Firmicutes decreased, while that of Bacteroidota and Proteobacteria increased in the ASG. However, no significant differences were detected between the two groups (*p* > 0.05).

Variations in the bacterial community at the genus level are presented in Figure 2B. *Lactobacillus* (47%), *Acetobacter* (26%), and *other genera* (16%). Relative to abnormal silage, relative to the CON group, the relative abundance of Lactobacillus and Corynebacterium in the ASG was decreased, while that of Prevotella, Acetobacter and other genera was increased.

### 3.3 Analysis of volatile metabolites in silage

#### 3.3.1 Analysis of sensory flavor characteristics in silage

**Figure 3:**
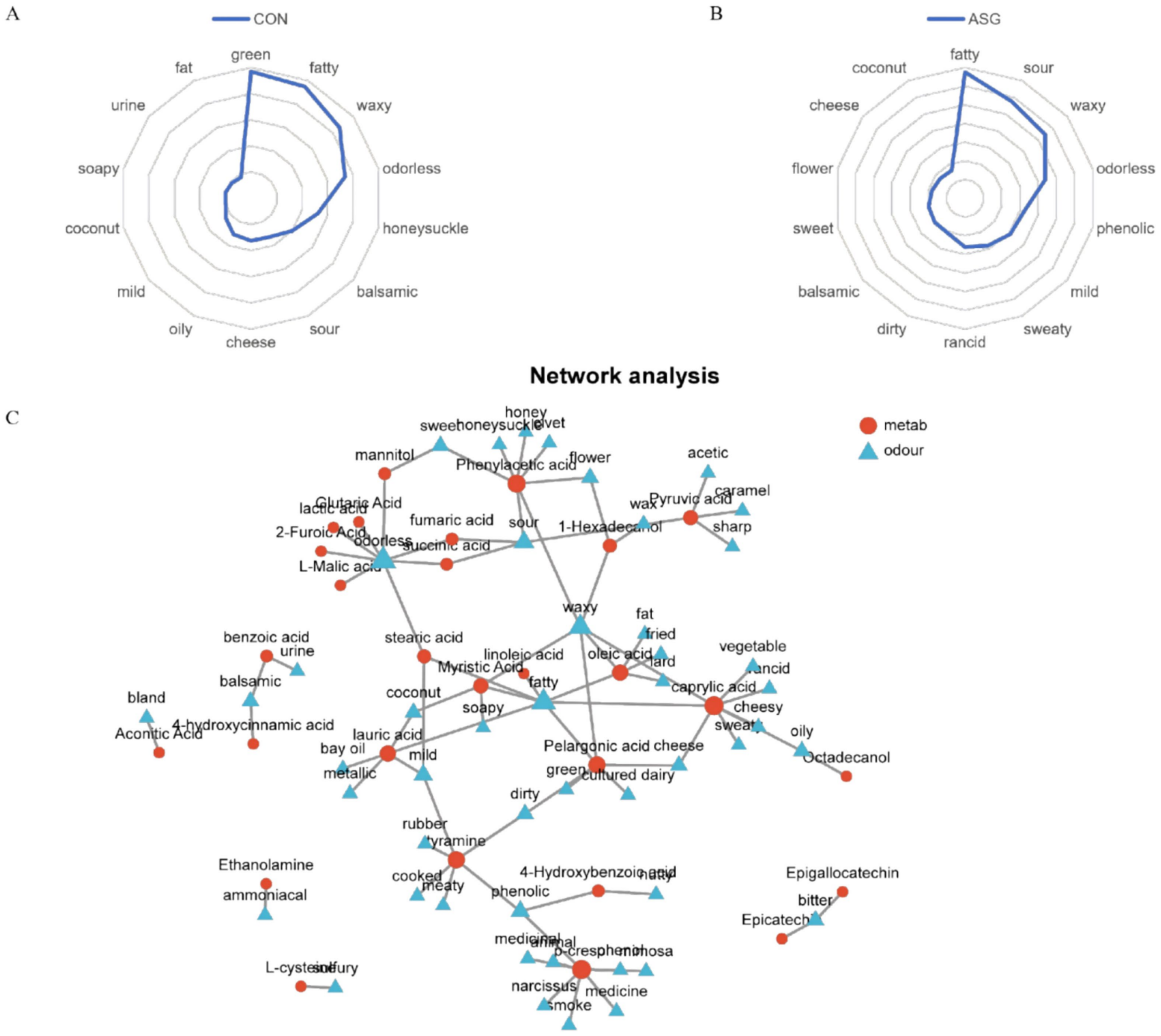
Radar chart showing the sensory flavor traits of distinct metabolites, along with a network graph that lays out the links between these sensory flavors and the same metabolites. **(A)** Sensory radar chart of CON, **(B)** sensory radar chart of ASG, **(C)** network diagram of associations between sensory flavors and differential metabolites. In **(C)**, blue circles are used to represent sensory characteristics, while red circles are designated for flavor compounds. The larger a blue circle is, the greater the number of flavor compounds connected to the corresponding sensory characteristic, indicating that the sensory characteristic is more significant. Correspondingly, the larger a red circle is, the higher the number of sensory characteristics linked to the target flavor compound, which reflects that the flavor compound has a more crucial role.

Sensory evaluation combined with differential metabolite analysis enabled systematic characterization of the sensory flavor traits of silage samples. As shown in Figures 3A,B, a radar chart was constructed to illustrate the top 15 sensory flavors. These flavors were selected based on the highest annotation abundance from the differential volatile metabolites of the two whole-plant corn silage groups, in accordance with the prescreening criteria. Obvious differences in aroma characteristics were found between the ASG and CON corn silages. The CON was enriched in green, fatty, and waxy notes, whereas the ASG was enriched in fatty and sour notes. Figure 3C presents a network diagram illustrating the associations between sensory flavors and their corresponding differential flavor metabolites. Specifically, fumaric acid, succinic acid, and L-malic acid were identified as key contributors to the sour attribute. Phenylacetic acid metabolites were directly associated with odor attributes including sour, sweet, honey, and waxy. Oleic acid, linoleic acid, caprylic acid, and pelargonic acid were simultaneously responsible for the fatty, fried, and cheesy attributes. Mannitol was correlated with sweetness, and 4-hydroxybenzoic acid was associated with the phenolic attribute.

#### 3.3.2 Preliminary screening of metabolites

**Figure 4:**
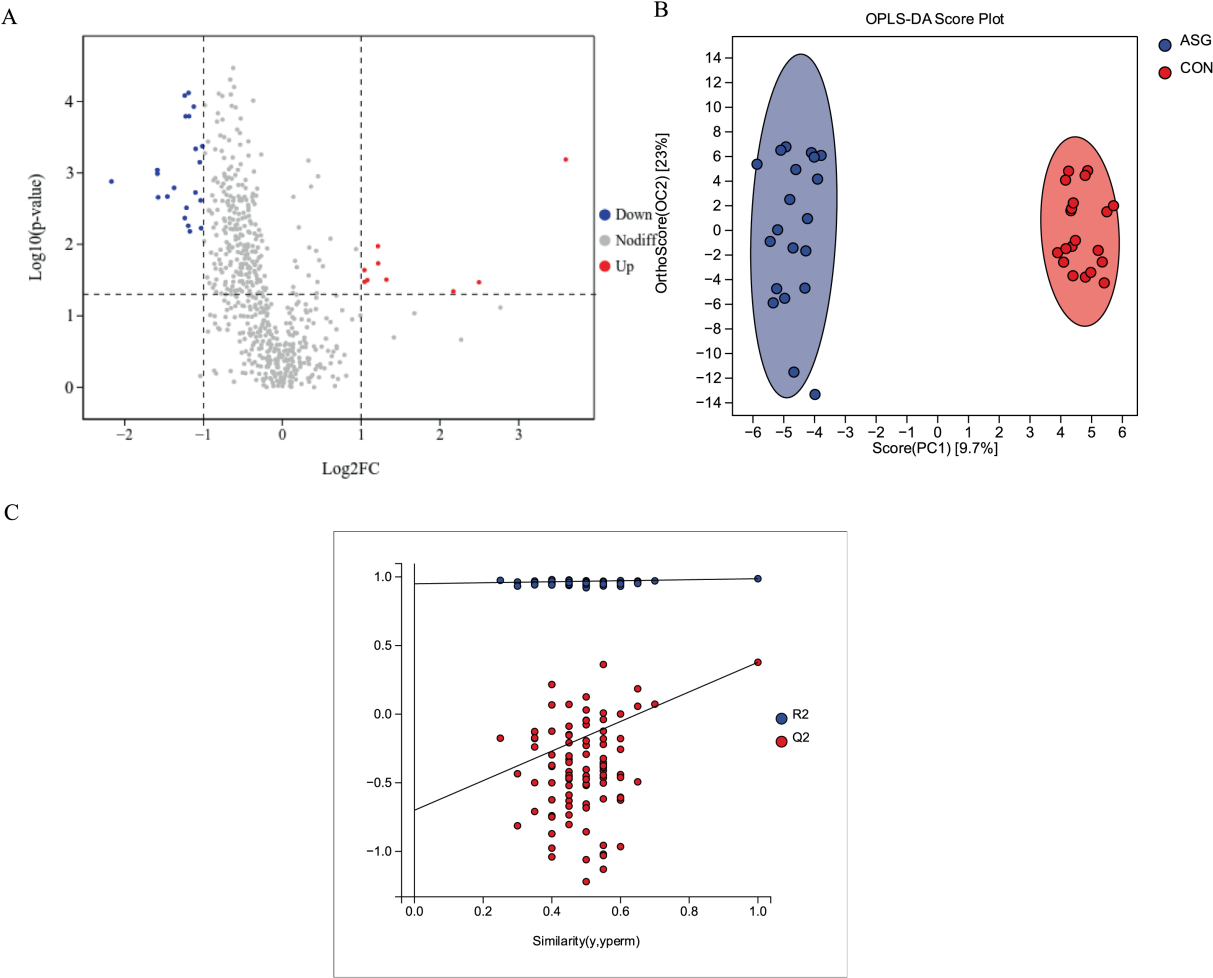
**(A)** Volcano plot illustrating the screening of differential metabolites, **(B)** OPLS-DA analysis, **(C)** Permutation test results of the OPLS-DA model.

As shown in Figure 4A, a total of 628 metabolites were identified in this study. Compared with the CON, 32 differentially abundant metabolites were detected in the ASG, of which 9 were upregulated and 23 were downregulated, while the remaining 596 metabolites showed no significant differences in their expression levels. To elucidate the differences in metabolites among silage samples at distinct fermentation stages, orthogonal partial least-squares discriminant analysis (OPLS-DA) was performed. Figure 4B shows a clear separation trend between the two silage groups. As an extension of PLS-DA, OPLS-DA was further applied for discriminant analysis (Figure 4C). The results indicated that the established OPLS-DA model showed no overfitting, exhibited favorable predictive ability and statistical significance, and could reliably distinguish samples from different groups.

#### 3.3.3 Secondary screening of metabolites in corn silage

**Figure 5:**
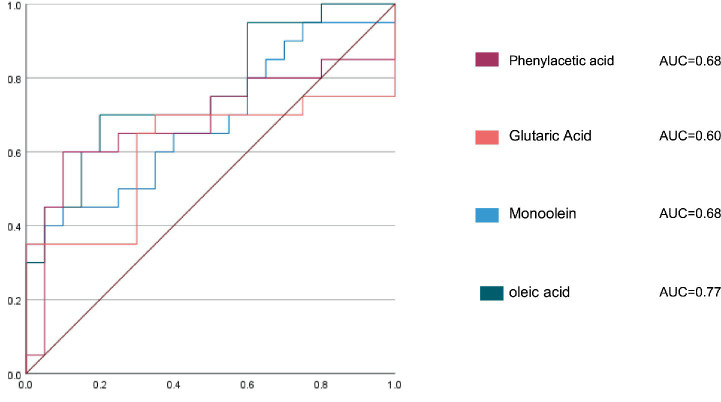
Receiver operating characteristic (ROC) analysis was used to assess the predictive accuracy of the models.

Based on the predefined screening criteria (FC >2 or <0.5, VIP > 1), qualified metabolites were sorted in ascending order of their values. Compared with the CON, eight metabolites were upregulated in the ASG, including methyl linoleate, α-sophorose, D-fructose, oleic acid, glyceryl monooleate, phenylacetic acid, glutaric acid, and trehalose. Six metabolites were downregulated in the ASG, namely glutaraldehyde 2, L-isoleucine, gallic acid, L-serine, 4-(hydroxymethyl)-3-methoxyphenoxyacetic acid, and 5′-cytidylic acid. As illustrated in Figure 5, the discriminative efficacy of phenylacetic acid and glutaric acid in classifying silage samples into the abnormal silage group (ASG, with off-flavor) and the control group (CON, without off-flavor) was validated using receiver operating characteristic (ROC) curve analysis. In the ROC curve analysis, oleic acid showed an area under the ROC curve value of 0.77 (95% CI: 0.62–0.91), indicating acceptable discriminatory power between the two silage groups. Glyceryl monooleate and phenylacetic acid both presented an area under the ROC curve value of 0.68 (95% CI: 0.52–0.85 and 0.50–0.86), suggesting moderate discriminative potential. By contrast, glutaric acid exhibited a low AUC value of 0.60 (95% CI: 0.42–0.79) with no statistical significance. Overall, oleic acid was identified as the optimal single metabolite biomarker for distinguishing the two silage groups with acceptable discriminatory power.

#### 3.3.4 Metabolic pathway enrichment analysis

**Figure 6:**
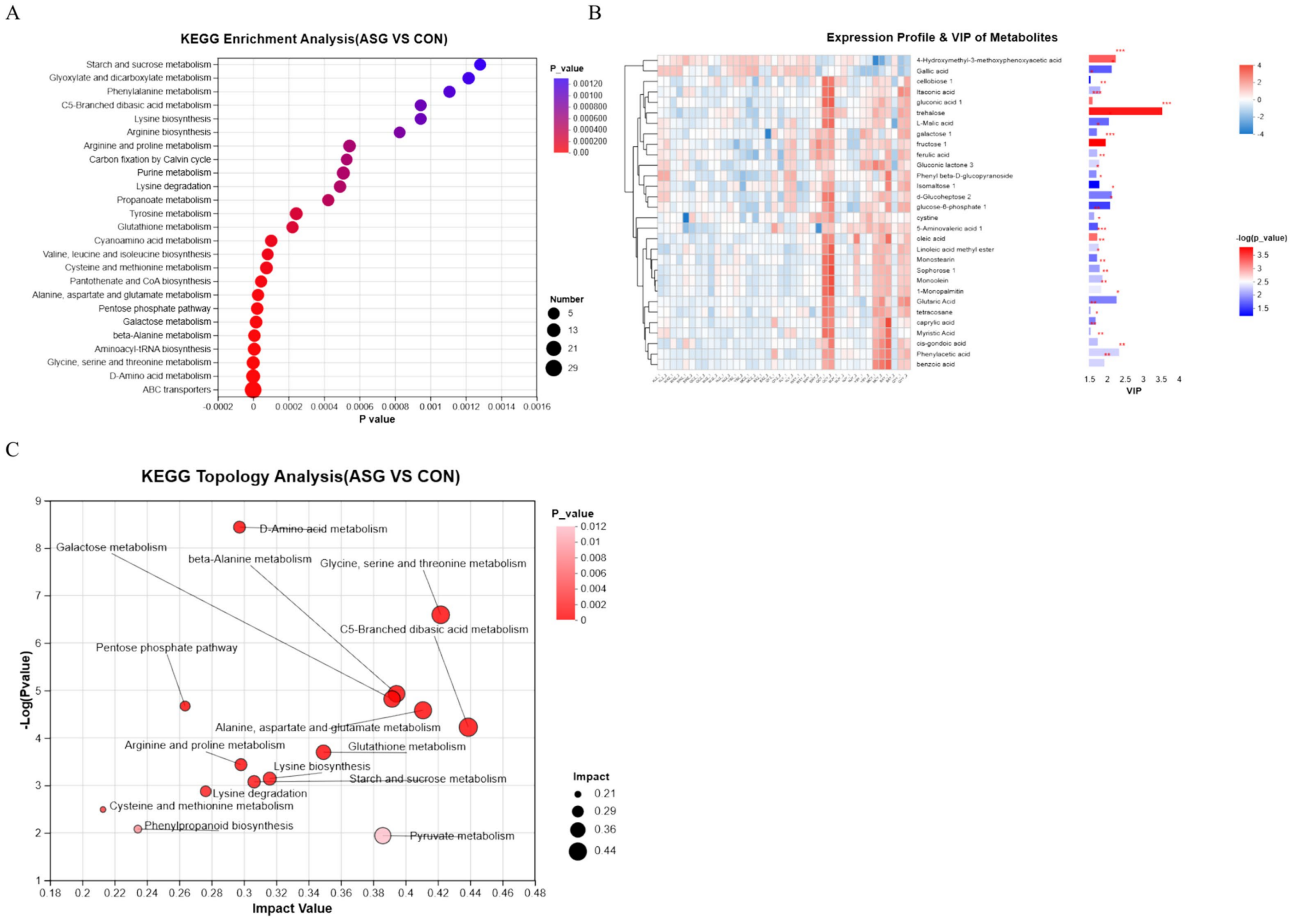
**(A)** Bubble plot of KEGG pathway enrichment analysis, **(B)** VIP analysis of differential metabolites in corn silage, **(C)** Bubble plot of KEGG pathway topological analysis.

As shown in Figure 6, KEGG metabolic pathway enrichment analysis was performed on the differential metabolites of whole-plant corn silage. The results of KEGG pathway enrichment analysis demonstrated that the ABC transporters pathway was highly significantly enriched between the CON and ASG. Additionally, enrichment of differential metabolites was also observed in pathways including starch and sucrose metabolism, bacterial chemotaxis, and biosynthesis of amino acids.

#### 3.3.5 Correlation analysis of silage microorganisms and key flavor compounds

**Figure 7:**
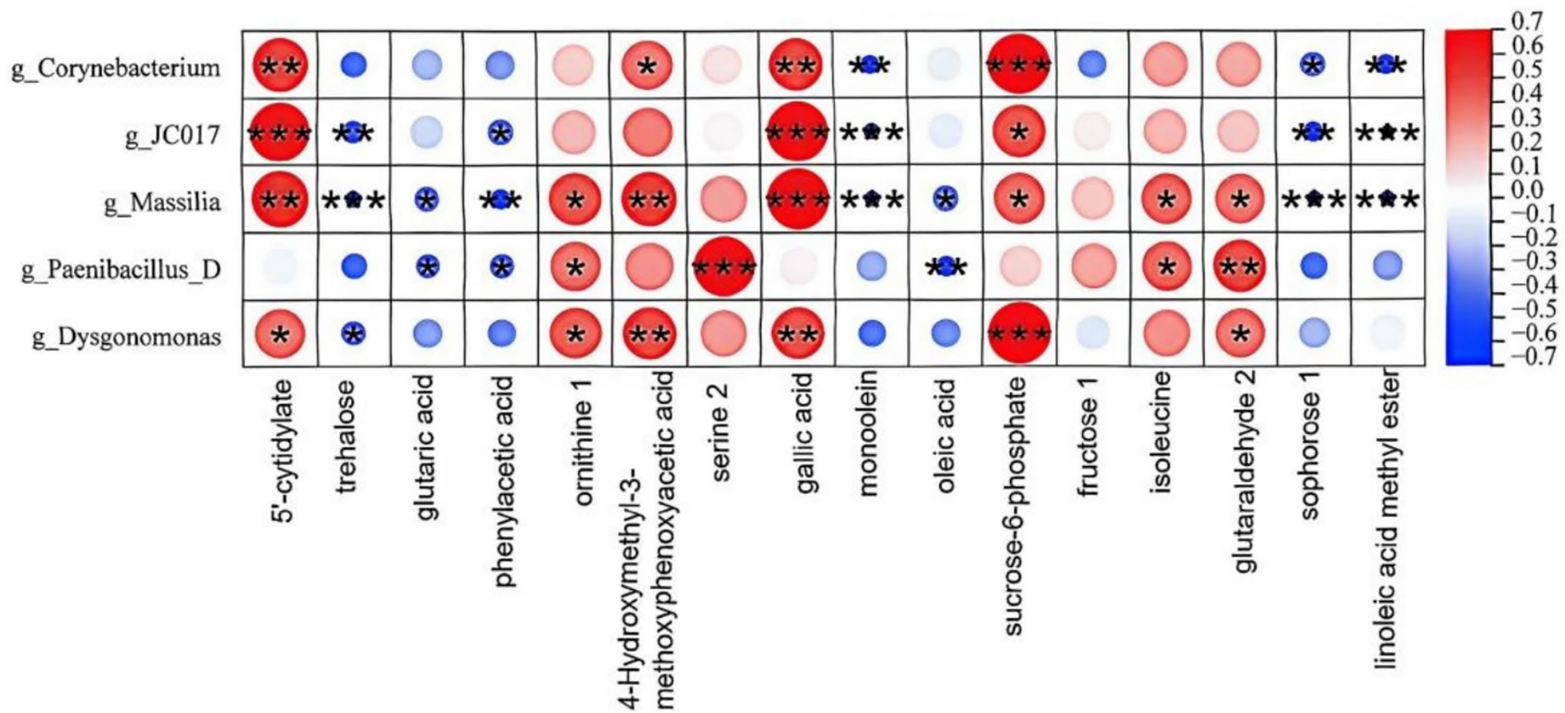
Correlation analysis between silage metabolomics and microbiome. This heatmap illustrates the correlations between microbial taxa and physicochemical factors, where color intensity indicates the strength of correlations. Asterisks (*) mark significantly associated “microbial taxa-physicochemical factor” pairs (*p* < 0.05).

To clarify the effects of microbial activity on flavor development during silage fermentation. we screened the top 40 major differential metabolites (VIP > 1 and *p*-value <0.05) to obtain 14 flavor metabolites (as presented in Table 2). As shown in Figure 7, Spearman correlation analysis was subsequently conducted to evaluate the associations between microorganisms and flavor metabolites. As shown in Figure 6, *g_Corynebacterium* was significantly positively correlated with glyceryl monooleate and Sucrose-6-Phosphate, but negatively correlated with 5′-cytidylate and glutaric acid (*p* < 0.05). *g_JC017* was strongly positively correlated with glyceryl monooleate and oleic acid, and negatively correlated with L-isoleucine and *α*-sophorose. *g_Massilia* was significantly positively correlated with 4-(hydroxymethyl)-3-methoxyphenoxyacetic acid and L-serine.

**Table 2:** Composition of differential metabolites in two types of silage.

Chemical taxonomy		Compounds	VIP	FC	Odor
Super class	Sub class				
Up-regulated metabolites					
Lipid molecules	Nitrite ester compounds	Linoleic acid methyl ester	1.24	2.06	At 100.00%. oily fatty woody peculiar lard-like
	Mono-unsaturated fatty acids	Oleic acid	1.64	2.32	Specific lard-like characteristics
	Monoacylglycerol compounds	Monoolein	1.35	2.32	Bland fatty-waxy texture
Organic oxygen compounds	Hexulose	Fructose 1	1.17	2.11	Odorless at 100.00% concentration
	Non-reducing disaccharides	Trehalose	1.82	12.10	Mild sweet taste
Benzene and substituted derivatives	Carboxylic acid compounds	Phenylacetic acid	1.43	2.50	Putrefactive fermentation-related compounds in honey
Organic acids and derivatives	Dicarboxylic acid compounds	Glutaric acid	1.53	4.50	Pungent taste
	Oligosaccharides	Sophorose 1	1.26	2.06	—
Down-regulated metabolites					
Nucleosides, nucleotides, and their analogs	Nucleotide	5′-cytidine monophosphate	1.63	0.50	Odorless
Organic acids and derivatives	Non-essential amino acids	Serine 2	1.19	0.44	Odorless with sweet taste
	Essential amino acids	Isoleucine	1.56	0.34	Odorless
Benzene and substituted derivatives	Polyphenols	Gallic acid	1.64	0.43	Odorless or weak odor
Organic oxygen compounds	Saturated straight-chain aliphatic dialdehydes	Glutaraldehyde 2	1.58	0.33	Pungent aldehyde odor
	Aromatic organic acids	4-Hydroxymethyl-3-methoxyphenoxyacetic acid	1.98	0.47	—

Flavor compounds refer to differential metabolites screened based on a *p*-value threshold of *p* < 0.05. The odor descriptions were derived from the following sources: https://www.chemicalbook.com/ShowSupplierProductsList1847976/0.htm, https://perflavory.com/, and relevant literature.

## 4 Discussion

### 4.1 Influence of off-flavor in silage on nutritional traits and fermentation quality

In this study, the pH value of silage in the ASG was 4.99, which was significantly higher than that in the CON (*p* < 0.01). This indicated that the acidic environment conducive to lactic acid fermentation had been disrupted, such that the proliferation of spoilage microorganisms could not be effectively inhibited, thereby inducing nutrient deterioration (Liu et al., 2024). A difference in the sampling positions between the two groups was identified as one of the key factors contributing to these changes: silage samples in the ASG were collected from the edge zones of the silo, whereas those in the CON were obtained from the core fermentation zones within the silo. Compared with the core fermentation zone, the edge zones of the silo are generally characterized by lower compaction density owing to their proximity to the silo wall, along with more significant temperature fluctuations. The fermentation quality of silage can also be influenced by numerous regional factors, such as temperature (Bernardes et al., 2018). As a result, air infiltration is likely to occur, which disrupts the anaerobic fermentation environment (Blajman et al., 2022; Liu et al., 2024; Sucu et al., 2016).

Compaction density exerts a significant influence on the fermentation characteristics of silage (Wang et al., 2017). Previous studies have demonstrated that with increasing compaction density, the pH value and ammonia nitrogen/total nitrogen (AN/TN) ratio of silage gradually decrease, while the lactic acid content shows an increasing trend (Liu et al., 2024; Tian et al., 2020). This indicates that higher compaction density is conducive to the formation of a favorable anaerobic environment and promotes lactic acid fermentation. In the present study, the ASG, collected from the silo edge areas with lower compaction density, exhibited a significant reduction in lactic acid content and a significant increase in pH value and AN/TN ratio, which is consistent with the conclusions of the aforementioned studies. Acetic acid plays an important preservative role in silage by inhibiting the growth of molds and harmful bacteria, thus enhancing storage stability (Danner et al., 2003). The higher acetic acid content observed in silage with a normal odor may reflect more complete fermentation by lactic acid bacteria, resulting in a more stable fermentation environment.

In terms of nutritional components, the silage in the ASG exhibited a significant decrease in dry matter (DM) content, while the contents of neutral detergent fiber (NDF) and acid detergent fiber (ADF) were significantly increased. The underlying reasons may be as follows: insufficient compaction density results in inadequate disruption of corn stover cells, preventing the effective release of intracellular nutrients. Meanwhile, the lower density increases the contact between silage materials and oxygen, thereby exacerbating aerobic metabolism and dry matter loss (Brüning et al., 2018). Additionally, the increased contents of NDF and ADF may be attributed to insufficient degradation of cellulose and hemicellulose by microorganisms during silage fermentation, which is consistent with the findings of Dean et al. (2005). In contrast, Liu et al. (2025) reported that the reduction in NDF and ADF contents stems from the utilization of cellulose and hemicellulose as fermentation substrates by microorganisms, which further confirms the inference in this study that abnormal fermentation in the ASG leads to increased fiber components. This finding is further supported by the elevated AN/TN ratio. AN/TN can be used to indicate the degree of protein degradation during silage fermentation. NH_3_-N is an indicator of protein degradation in silage (Ohshima and McDonald, 1978), with higher concentrations reflecting more severe protein breakdown, which accounts for the lower ammonia nitrogen and crude protein contents in the ASG. Butyric acid has a strong rancid butter odor, which impairs silage palatability and aggravates energy loss and protein degradation simultaneously (Krízek, 1993; Scherer et al., 2019).

### 4.2 Effects of silage off-flavors on microbial community structure

The structural dynamics and functional shifts of microbial communities during silage fermentation constitute the core mechanism regulating flavor quality (Rajer et al., 2017; Raza et al., 2016). Essentially, this represents a dynamic equilibrium process involving nutritional competition and metabolite interactions between core functional bacteria and spoilage microorganisms (Weinberg, 2008; Yin et al., 2023). In the present study, the α-diversity of the ASG was significantly higher than that of the CON (*p* < 0.05). No significant separation was observed between the two groups in PCoA analysis(as shown in Figure 1B), with within-group variation exceeding between-group variation. This may be attributed to the excessive proliferation of harmful microorganisms, which gave rise to specific taxa and disrupted the original microbial balance, thereby driving the fermentation pattern from a lactic acid-dominated type toward a putrefactive type. Silage fermentation involves the synergistic action of diverse microorganisms, whose interactions directly determine silage quality and nutritional value (Li et al., 2023).

In the CON group, Lactobacillus dominated homolactic fermentation at a high relative abundance of 47%, thereby establishing an acidic microenvironment that suppressed spoilage microorganisms (Huang et al., 2021). This observation is highly consistent with the regulatory framework of “lactobacilli-lactic acid-acid inhibition” proposed in classic silage fermentation theory (Ogunade et al., 2018). In contrast, the abundance of Lactobacillus was markedly decreased in the ASG, accompanied by a reduced proportion of Firmicutes and the enrichment of Proteobacteria and Bacteroidota. As a result, the lactic acid concentration was reduced from 5.3 to 4.27, and the microecological balance of the fermentation system was disrupted. This phenomenon is consistent with the conclusion reported by Robinson and Swanepoel (2016) that oxygen exposure induces microbial spoilage in silage, verifying that oxygen intrusion is a key trigger for microbial community imbalance. Dong et al. (2023) confirmed that prolonged contact between silage and air results in lower populations of lactic acid bacteria. Keshri et al. (2018) demonstrated that inoculation with Lentilactobacillus buchneri can reduce butyric off-odors and enhance fruity sweetness. Wilkinson and Wilkinson and Davies (2013) pointed out that long-term aerobic exposure induces secondary fermentation, inhibits the proliferation of lactobacilli, and leads to the deterioration of silage quality. From the perspective of microbial interactions, lactic acid bacteria are considered to gain a competitive nutritional advantage through the rapid utilization of soluble carbohydrates. The lactic and acetic acids produced during their metabolism lower the environmental pH, thereby inhibiting the proliferation of acid-intolerant spoilage microorganisms (Feng et al., 2023). In follow-up studies, we will integrate mult-omics analysis with molecular biology validation to enable deeper exploration of the core mechanisms. Overall, this study remains exploratory, and further research is needed to fully elucidate the core mechanisms.

### 4.3 Effects of flavor compounds on silage

The results of sensory evaluation revealed that normal silage (CON) was significantly enriched in flavor dimensions such as grassy, fatty, and waxy notes, whereas off-flavor silage (ASG) was characterized predominantly by fatty and sour tastes. In the analysis of key off-flavor compounds, the butyric acid content in the ASG was observed to be abnormally elevated to 2.10%, compared with 0.02% in the CON. This compound, characterized by a strong rancid butter-like odor, is considered one of the core contributors to silage flavor deterioration (Kumar and Chand, 2015). Previous studies have demonstrated that the accumulation of butyric acid is directly associated with the proliferation of butyric acid-producing bacteria, such as those of the genus Clostridium (Danner et al., 2003). In the present study, the microenvironment of the ASG, with pH elevated to 4.99, was found to provide favorable conditions for the active metabolism of these bacteria, thereby further validating the regulatory pathway linking functional microbiota and off-flavor compounds. Meanwhile, significant upregulation of unsaturated fatty acids, including oleic acid and linoleic acid, was observed in the ASG.

In the ASG, a significant upregulation of oleic acid content was observed, suggesting its potential as a metabolic marker for distinguishing between normal and off-flavor silage. Through sensory-flavor association analysis, oleic acid was further revealed to be closely related to sensory attributes such as fatty, fried, and cheesy notes, and its accumulation may directly contribute to the characteristic oily and rancid off-flavor profile of the ASG. From the perspective of microbial metabolism, a significantly strong positive correlation was found between oleic acid content and the abundance of genus JC017. Although the function of genus JC017 in this study remains unclear, its positive association with unsaturated fatty acids implies that this genus may be involved in lipid metabolism or may have a synergistic relationship with oleic acid-producing microorganisms. Under aerobic exposure conditions, unsaturated fatty acids such as oleic acid are susceptible to hydrolysis and oxidation mediated by microbial lipases (Jenkins et al., 2008). As a result, volatile secondary products, including short-chain fatty acids, aldehydes, and ketones, are generated (Jenkins et al., 2008), which in turn induce the rancid off-flavor characteristic of oil oxidation. Oleic acid itself is not a typical off-flavor compound and is present at a certain level in normal silage. However, when microbial dysbiosis occurs in the silage system, accompanied by an increase in pH, the metabolic fate of oleic acid is altered: it is released from its stable esterified form into free fatty acids, which are then oxidatively degraded into volatile off-flavor products (Shahidi and Hossain, 2022). Therefore, the upregulation of oleic acid may reflect an intensification of lipid hydrolysis and oxidation processes in silage, rather than a direct contribution to the odor. This mechanism is consistent with the observations of Khan et al. (2009) in grass silage, where a significant decrease in unsaturated fatty acid content was detected after aerobic exposure, accompanied by the generation of volatile oxidation products.

Phenylacetic acid (PAA) was found to be significantly upregulated in the ASG. This compound exhibits a concentration-dependent dual odor profile: honey-like sweetness at low concentrations, but rancid honey and animal-like mustiness at high concentrations, making it an off-flavor marker for distinguishing between the two silage groups. In the study by Barata et al. (2011), it was demonstrated that wines produced from sour rotten grapes exhibited a pronounced honey-like aroma, whereas this characteristic was not observed in wines produced from healthy grapes. Phenylacetic acid and ethyl phenylacetate were identified as the key aroma compounds of sour rotten grape wines. Based on the results of Spearman correlation analysis and KEGG pathway enrichment, the abundance of *Corynebacterium* in the ASG was significantly decreased and negatively correlated with phenylacetic acid content. Consequently, it is hypothesized that *Corynebacterium glutamicum*, a representative species of the genus *Corynebacterium*, together with its closely related species, plays a key role in the silage microbial ecosystem. These corynebacteria are considered to possess adaptive characteristics, including acid tolerance and facultative anaerobiosis, and are capable of effectively metabolizing phenylacetic acid (Chen et al., 2012). This result is consistent with the hypothesis that the reduced abundance of *Corynebacterium* observed in this study may lead to a disruption of the subsequent phenylacetic acid transformation pathway mediated by this genus, consequently contributing to the upregulation of phenylacetic acid and the generation of undesirable odors in silage.

It is noteworthy that the flavor quality of silage is not determined by a single compound but arises from synergistic interactions among multiple metabolites. In the ASG, the simultaneous accumulation of off-flavor compounds, including butyric acid, phenylacetic acid, and oleic acid, together with a marked decrease in positive flavor compounds such as lactic acid and acetic acid, collectively shaped the characteristic flavor profile of off-flavor silage. Based on the multi-omics data from this study, we systematically elucidated a three-level correlation network linking microbial community succession, metabolite profile shifts, and sensory flavor traits, providing a robust theoretical basis for the targeted regulation of silage flavor quality.

### 4.4 Key metabolic pathways and microbial associations in silage flavor formation

The generation and accumulation of flavor compounds in silage result from the synergistic effects of microbial community metabolic regulation and substrate utilization, with the interaction mechanisms between core pathways and functional microorganisms being key to elucidating the deterioration of flavor quality (Zhang et al., 2021). Through KEGG enrichment analysis of differential metabolites, the formation of off-flavor in corn silage was found to be closely associated with the dysregulation of four core pathways: ABC transporter, starch and sucrose metabolism, phenylalanine metabolism, and fatty acid metabolism. These pathways were revealed to form clear regulatory links with the abundance changes of key microbial taxa, thereby providing direct clues for understanding the microecological mechanisms underlying flavor compound formation.

At the level of carbohydrate metabolism, the disruption of starch and sucrose metabolism was identified as a core driver underlying the imbalance of fundamental flavor substrates. In the control group, the high abundance of Lactobacillus ensured efficient homolactic fermentation, through which carbohydrates were rapidly converted into lactic acid, thereby maintaining a low pH environment and suppressing the proliferation of spoilage microorganisms (Mu et al., 2020). This process was closely associated with the pronounced enrichment of the ABC transporter pathway, which is responsible for the transmembrane transport of carbohydrates and antimicrobial metabolites (Jeckelmann and Erni, 2020; Xie et al., 2018). The dysregulation of this pathway may further exacerbate the imbalanced allocation of nutrient substrates and the abnormal accumulation of metabolites, thereby serving as a pivotal hub linking the succession of microbial communities to the alteration of flavor compounds.

In terms of the formation of aromatic and lipid-derived flavor compounds, the regulatory roles of the phenylalanine metabolism and fatty acid metabolism pathways were particularly prominent. Based on the results of KEGG pathway enrichment analysis, *Corynebacterium glutamicum* was indicated to potentially inhibit the accumulation of phenylacetic acid through its involvement in the phenylalanine catabolic pathway. In this pathway, phenylacetic acid is further degraded into acetyl-CoA and succinyl-CoA by the enzyme system encoded by the paa gene cluster (Teufel et al., 2010). When the abundance of *Corynebacterium* is reduced, the metabolic activity of this degradation pathway is attenuated, leading to the accumulation of phenylacetic acid. Meanwhile, the abundance of genus *JC017* was shown to be strongly positively correlated with unsaturated fatty acids such as oleic acid and linoleic acid. These compounds are subjected to oxidative degradation mediated by microbial lipases, generating small-molecule flavor compounds including aldehydes and ketones, which ultimately contribute to the sensory characteristics of rancidity and greasiness. Furthermore, the positive correlation observed between *Paenibacillus_D* and the antimicrobial metabolite gallic acid revealed a regulatory loop involving beneficial bacteria, antimicrobial metabolites, and fermentation stability. Depletion of gallic acid was suggested to have weakened the inhibition of spoilage microorganisms, thereby amplifying the negative impact of aberrant metabolic pathways on flavor quality (He et al., 2020). Note that CON and ASG samples were taken from silo centers and edges, respectively, so off-flavor was confounded with sampling position. Differences in nutrition, microbial community, and metabolites may stem from positional factors rather than off-flavor alone. This is a design limitation. Future controlled studies are required to distinguish these effects.

It should be noted that, as 16S rRNA sequencing reflects only microbial community structure, associations between metabolic pathways and microorganisms were primarily inferred from correlation analyses and existing theoretical support. To validate these hypotheses, *in vitro* metabolite validation will be performed, including Corynebacterium culture with exogenous addition of phenylacetic acid or phenylalanine. Substrate consumption and product formation will be assessed by HPLC or mass spectrometry, and expression of key genes (e.g., the paa gene cluster) will be examined by qPCR. Overall, the potential regulatory relationships among core microbial communities, key metabolic pathways, and flavor compounds in ensiled maize were preliminarily delineated, providing a reference for understanding the microecological mechanisms underlying silage flavor formation and offering a theoretical and practical basis for the targeted management of feed quality, production performance, milk flavor, and disease control in dairy cattle.

## 5 Conclusion

This study found that the decrease in Lactobacillus abundance in off-flavor silage may be associated with reduced lactic acid accumulation and elevated pH, accompanied by dysregulation of butyrate, phenylalanine, and fatty acid metabolism. These changes potentially contributed to the accumulation of off-flavor compounds (e.g., butyric acid, phenylacetic acid, oleic acid) and depletion of antimicrobial substances (e.g., gallic acid), ultimately driving a shift from lactic acid-dominated to spoilage fermentation. Based on this, a cascade hypothesis of “decrease in Lactobacillus count-accumulation of off-flavor substances-flavor deterioration” was preliminarily validated, and a regulatory network of “core microbiota-key metabolic pathways-flavor compounds” was constructed, providing a theoretical basis for the targeted microbial regulation of silage quality and the implementation of early warning mechanisms during the production process.

## Data Availability

The data presented in the study are deposited in the NCBI SRA repository, accession number PRJNA1455070.

## Footnotes

1
https://www.genescloud.cn/home
