# Supplementary material for: Impacts of off-flavor on microbial community structure, nutritional traits and metabolite profiles in whole-plant corn silage
Source: Front Microbiol. 2026 Apr 30;17:1827817. doi: 10.3389/fmicb.2026.1827817 (PMC13200608; doi:10.3389/fmicb.2026.1827817)
Supplement: Supplementary file 1 [file Table_1.DOCX]

Table 1. Sensory Evaluation of Silage

| **Sensory Indicators** | **Scoring Criteria** | | | **Score** |
| --- | --- | --- | --- | --- |
| Oder | Strong or distinct bread aroma or distinct bread aroma, no but uric acid odor. | | | 14 |
|  | Faint but uric acid odor, or strong acidity, weak aroma. | | | 10 |
|  | Thick but uric acid odor, pungent burnt odor, or musty odor. | | | 4 |
|  | Strong but uric acid or ammonia odor, almost no acid. | | | 2 |
| Structure | Stem and leaf structure are intact and clearly visible. | | | 4 |
|  | Stem and leaf structure are slightly damaged. | | | 2 |
|  | Stem and leaf structure are damaged obviously or slightly contaminated. | | | 1 |
|  | Stem and leaf rotted or seriously contaminated. | | | 0 |
| Color | Close to raw material color, light brown after drying. | | | 2 |
|  | Slight discoloration, light yellow or green and yellow. | | | 1 |
|  | Severe discoloration, dark green or brown. | | | 0 |
| Total score | 16～20 | 10～15 | 5～9 | 0～4 |
| Grade | Grade 1 (Excellent) | Grade 2 (Good) | Grade 3 (Medium) | Grade 4 (Rotten) |

Table 2**.** Differential analysis of microbial phylum and genus levels in silage

| Taxon | CON | ASG | P-value |
| --- | --- | --- | --- |
| g_Lactobacillus | 0.47 | 0.27 | <0.05 |
| g_Acetobacter | 0.26 | 0.34 | <0.05 |
| Others | 0.16 | 0.22 | >0.05 |

| Taxon | CON | ASG | P-value |
| --- | --- | --- | --- |
| p_Firmicutes_D | 0.54 | 0.38 | >0.05 |
| p_Proteobacteria | 0.35 | 0.46 | >0.05 |
| p_Actinobacteriota | 0.06 | 0.05 | >0.05 |
| p_Bacteroidota | 0.02 | 0.06 | >0.05 |
